# Earliest observation of the tetracycline destructase *tet(X3*)

**DOI:** 10.1128/spectrum.03327-23

**Published:** 2024-02-27

**Authors:** Frédéric Grenier, Simon Lévesque, Sébastien Rodrigue, Louis-Patrick Haraoui

**Affiliations:** 1 Department of Biology, Université de Sherbrooke, Sherbrooke, Québec, Canada; 2 Department of Microbiology and Infectious Diseases, Université de Sherbrooke, Sherbrooke, Québec, Canada; 3 CIUSSS de l’Estrie - CHUS, Sherbrooke, Québec, Canada; 4 Centre de recherche Charles-Le Moyne, Greenfield Park, Québec, Canada; University at Albany, Albany, New York, USA; Umeå University, Umeå, Sweden; Universiteit van Amsterdam, Amsterdam, The Netherlands

**Keywords:** antimicrobial resistance, tetracycline destructase, *tet(X3)*, *bla*_OXA-58_, *Acinetobacter junii*, Israel

## Abstract

Tigecycline is an antibiotic of last resort for infections with carbapenem-resistant *Acinetobacter baumannii*. Plasmids harboring variants of the tetracycline destructase gene *tetX* promote rising tigecycline resistance rates. We report the earliest observation of *tet(X3*) in a clinical strain predating tigecycline’s commercialization, suggesting selective pressures other than tigecycline contributed to its emergence.

**IMPORTANCE:**

We present the earliest observation of a *tet*(*X3*)-positive bacterial strain, predating by many years the earliest reports of this gene so far. This finding is significant as tigecycline is an antibiotic of last resort for carbapenem-resistant *Acinetobacter baumannii* (CRAB), which the World Health Organization ranks as one of its top three critical priority pathogens, and *tet*(*X3*) variants have become the most prevalent genes responsible for enabling CRAB to become tigecycline resistant. Moreover, the *tet*(*X3*)-positive strain we report is the first and only to be found that predates the commercialization of tigecycline, an antibiotic that was thought to have contributed to the emergence of this resistance gene. Understanding the factors contributing to the origin and spread of novel antibiotic resistance genes is crucial to addressing the major global public health issue, which is antimicrobial resistance.

## OBSERVATION

Antimicrobial resistance (AMR) is a major global public health issue, with AMR deaths surpassing HIV and malaria (1). The World Health Organization (WHO)’s list of critical drug-resistant pathogens—*Acinetobacter baumannii*, *Pseudomonas aeruginosa,* and *Enterobacteriaceae—*share a common trait: resistance to carbapenems (2). Therapeutic options for carbapenem-resistant *Acinetobacter baumannii* are often limited to tigecycline and colistin. Resistance to these antibiotics is increasing, primarily due to *tet(X*) and *mcr* variants, respectively. *tet(X3*), the predominant *tet(X*) variant among *Acinetobacter* spp., was initially reported in 2019 in an *A. baumannii* isolated in China in 2017 (3). Retrospective analyses have since highlighted the role of non-*baumannii Acinetobacter* in the global distribution of *tet(X3*), with the earliest clinical isolates dating back to 2010 (4–6). We report the earliest observation of *tet(X3*) in an *Acinetobacter junii* strain (Ajun-H1-2) isolated from a blood culture in 2004 in Israel.

We obtained 198 clinical *Acinetobacter* spp. isolated in Israel between 2001 and 2006 from three archives: (i) Chaim Sheba Medical Center (CSMC), Tel HaShomer, Israel (*n* = 140); (ii) JMI Laboratories (*n* = 37 isolated at CSMC, distinct from (i); and (iii) *n* = 21 from International Health Management Associates, collected from two anonymized hospitals.

All isolates were grown in lysogenic broth at 37°C overnight. DNA libraries were prepared from extracted gDNA using the NEBNext Ultra II FS DNA Library Prep Kit for Illumina (NEB). DNA was purified and size selected using Ampure XP beads (Beckman Coulter) and quantified using Quant-it PicoGreen dsDNA assay (Thermo Fisher). Library quality and size distribution were assessed on a Fragment Analyzer using the HS NGS Fragment Kit (Agilent). The pooled samples were then sequenced on a NovaSeq6000 (Illumina) with 250 bp paired-end sequencing.

A subset of strains, including Ajun-H1-2, was also sequenced using an Oxford Nanopore Technologies (ONT) R10.4 Flow Cell on a MinION Mk1B. The extracted gDNA was treated with the NEBNext Ultra II End Repair/dA-Tailing Module (NEB). Barcodes from the Native Barcoding Expansion 1–12 and 13–24 from ONT were ligated using the NEBNext Ultra II Ligation Module (NEB). DNA was purified using Ampure XP beads (Beckman Coulter). The DNA from different barcoded samples was pooled, and the adapter AMII (ONT) was ligated using the NEBNext Ultra II Ligation Module (NEB).

For Illumina reads, quality assessment and trimming were done using fastp 0.21.0 with --cut_right --cut_window_size 4 --cut_mean_quality 20 --length_required 30 --detect_adapter_for_pe (7). Assemblies were made using Unicycler 0.4.9 (8) with the trimmed Illumina short reads and ONT long reads when available. Contigs were filtered to retain only those above 500 bp. Sequencing reads are deposited in GenBank as JANVQZ000000000.1 under BioProject number PRJNA845765.

Taxonomic identification was made on the assemblies using Kraken 2 (2.0.9-beta) (9). Antibiotic resistance genes (ARGs) were searched using ResFinder 4.1 (10). Detected *bla*_OXA_ variants were curated using BLDB (11). Assemblies were annotated with Prokka 1.14.5 (12) using additional databases (Pfam, TIGRFAM, and BLDB). To search and annotate plasmids’ replication and mobility types, we used PlasmidFinder 2.1 and MOB-suite 3.1.7 (13–15). The plasmid figure was generated using AliTV (16).

**Fig 1:**
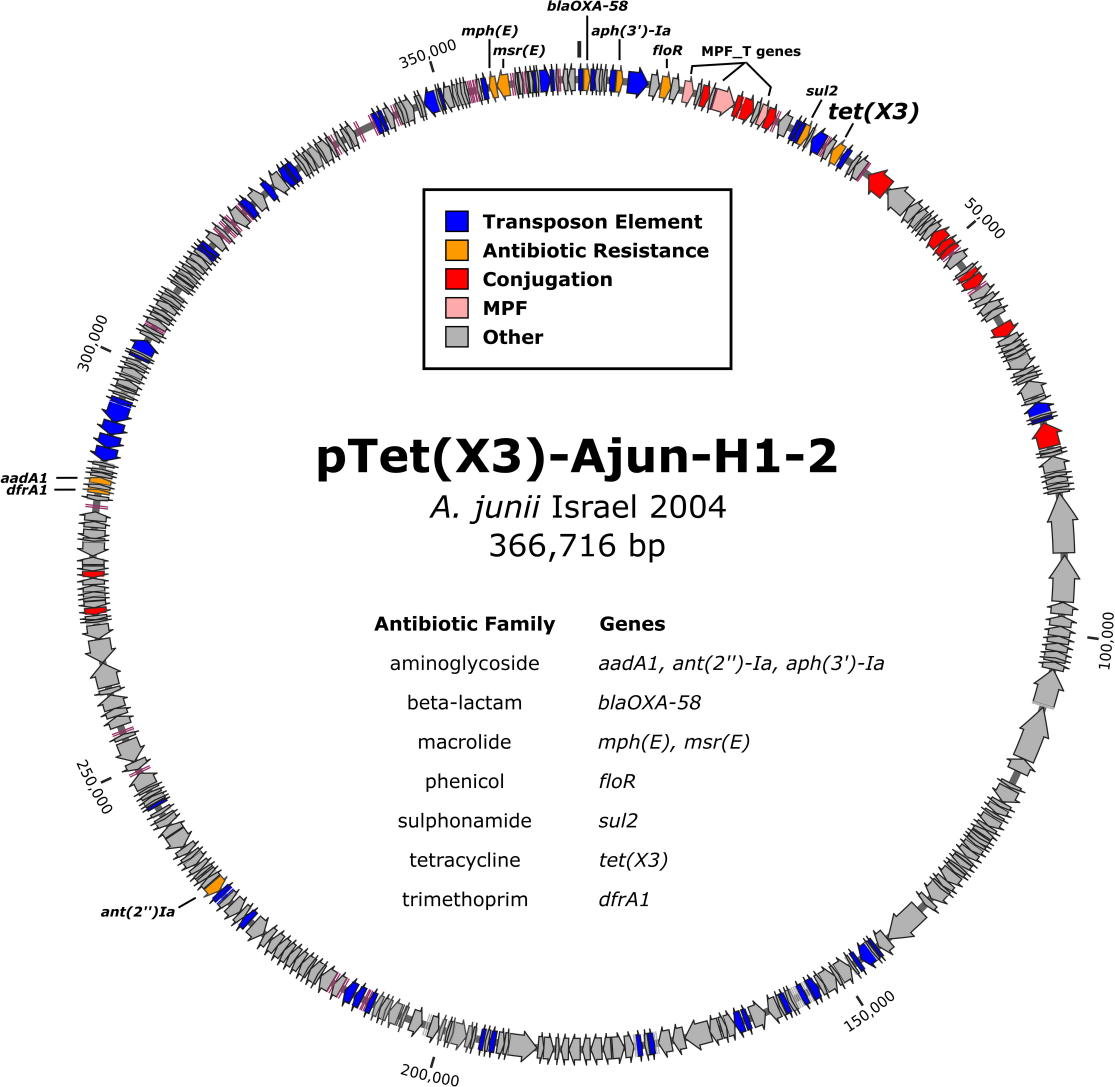
Representation and partial annotation of pTet(X3)-Ajun-H1-2 including all antibiotic-resistance genes identified and the mating pair formation type T genes.

An *A. junii* strain, isolated from a blood culture in 2004 in Israel and named Ajun-H1-2, carried several ARGs, including the *tet(X3*) tetracycline destructase and the *bla*_OXA-58_ carbapenemase. No further information about the patient from which it was isolated is available. All ARGs were located on a 367-kb plasmid named pTet(X3)-Ajun-H1-2. No origin of replication was identified. The output from MOB-suite did, however, reveal the presence of genes for mating pair formation (MPF) type T. Figure 1 presents partial annotation of pTet(X3)-Ajun-H1-2 including all ARGs and MPF_T genes. Strain Ajun-H1-2 also contained four other plasmids ranging in size between 2 and 8 kb.

**Fig 2:**
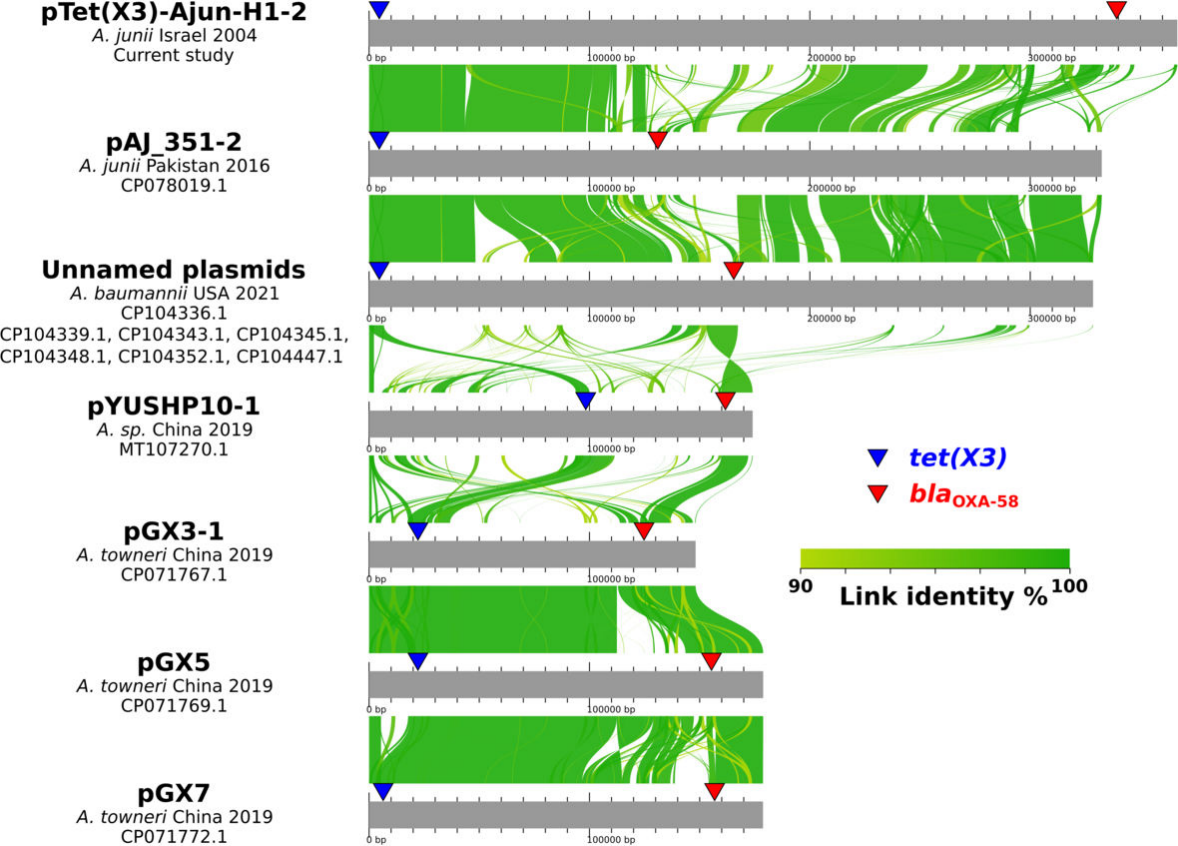
Alignments of 13 plasmids harboring both *tet(X3*) and *bla*_OXA-58_ including pTet(X3)-Ajun-H1-2 reported in this study. Alignments were done using AliTV (10).

Twelve other cases of *tet(X3*) and *bla*_OXA-58_ co-existence on the same plasmid have been reported among more recently isolated *Acinetobacter* spp. from healthcare settings in the United States (seven *A*. *baumannii*) and Pakistan (one *A*. *junii*), as well as from farm animals in China (three *Acinetobacter towneri* and one *Acinetobacter* spp.) (6, 17, 18). pTet(X3)-Ajun-H1-2 shares parts of its sequence with 8 of these 12 plasmids: pAJ_351-2 isolated in Pakistan in 2016 (*A. junii*), and seven unnamed plasmids isolated in the USA in 2021 (all in *A. baumannii*) (Fig. 2).

Antibiotic susceptibility testing was carried out with 19 antibiotics using the AST-N801 card on a Vitek2 (bioMérieux) and the Sensititre GNX3F plate (ThermoFisher). Ajun-H1-2 demonstrated decreased susceptibility (intermediate or resistant) to ciprofloxacin, colistin, gentamicin, tetracycline, tobramycin, and trimethoprim/sulfamethoxazole with varying minimal inhibitory concentrations depending on the method used. The strain was sensitive to other antibiotics, including tigecycline, doxycycline, minocycline, as well as all beta-lactams (Table 1).

**TABLE 1:** Antibiotic susceptibility results obtained using the AST-N801 card on a Vitek2 and a Sensititre GNX3F plate^*a*^

Antibiotic	Vitek MIC	Vitek interpretation	Sensititre MIC	Sensititre interpretation
Tetracycline	≥16	R		
Doxycycline			4	S
Minocycline			2	S
Tigecycline	1	S	2	S
Ampicillin/sulbactam	≤2	S	4/2	S
Ticarcillin/clavulanic acid			16/2	S
Piperacillin/tazobactam	≤4	S	8/4	S
Ceftazidime	≤1	S	2	S
Ceftolazone/tazobactam	≤0.25	S		
Cefepime	0.5	S	2	S
Imipenem	≤0.25	S	1	S
Meropenem	≤0.25	S	1	S
Doripenem			0.5	S
Ciprofloxacin	2	I	2	I
Levofloxacin	2	S	1	S
Amikacin	≤2	S	4	S
Gentamicin	≥16	R	8	I
Tobramycin	≥16	R	8	I
Trimethoprim/sulfamethoxazole	≥320	R	4/76	R

^*a*^ MIC, minimal inhibitory concentration.

This discrepancy between *tet(X3*) presence and resistance profile has been primarily noted in strains isolated in farm animals. In one report of 47 *tet(X3*)-positive *Acinetobacter* strains, only one showed an intermediate susceptibility to tigecycline, and four were found to be resistant to doxycycline and minocycline (tetracycline was not assessed) (19). Such discrepancy could be related to *tet(X3*) variants present (20) or also to *tet(X3*) copy number (3). Indeed, strain AB34 (3) carried three copies of *tet(X3*) and was resistant to all major tetracycline antibiotics, whereas the Ajun-H1-2 strain we describe only carried one copy of *tet(X3*).

### Conclusion

Our report adds further clue on the role of non-*baumannii Acinetobacter* in the initial dissemination of *tet(X3*). Whereas the use of tigecycline has been linked to the rise of *tet(X*) variants, this study demonstrates that *tet(X3*) predated the commercialization of this antibiotic in 2005.

Although cases of *tet(X3*)-positive *Acinetobacter* spp. have been isolated on nearly all continents (4, 21), most reports come from China, which may reflect sampling and reporting biases. Tetracycline antibiotics are widely used in various settings in China, including in animal husbandry and other agricultural practices (22), which could also explain a greater number of reported cases.

Finally, we highlight the limitations of relying on antibiotic susceptibility testing as a means of retrospectively tracking the emergence and spread of ARGs. Further research is needed to more fully understand the origins of *tet(X3*) as well as minimal inhibitory concentration variations among *tet(X3*)-positive strains.

## Supplementary Material

Reviewer comments

## SUPPLEMENTAL MATERIAL

OPEN PEER REVIEW reviewer-comments.pdf An accounting of the reviewer comments and feedback.

ASM does not own the copyrights to Supplemental Material that may be linked to, or accessed through, an article. The authors have granted ASM a non-exclusive, world-wide license to publish the Supplemental Material files. Please contact the corresponding author directly for reuse.

## References

[1] Murray CJL, Ikuta KS, Sharara F, Swetschinski L, Robles Aguilar G, Gray A, Han C, Bisignano C, Rao P, Wool E, et al.. 2022. Global burden of bacterial antimicrobial resistance in 2019: a systematic analysis. Lancet 399:629–655. doi:10.1016/S0140-6736(21)02724-0 35065702 PMC8841637

[2] Tacconelli E, Carrara E, Savoldi A, Harbarth S, Mendelson M, Monnet DL, Pulcini C, Kahlmeter G, Kluytmans J, Carmeli Y, Ouellette M, Outterson K, Patel J, Cavaleri M, Cox EM, Houchens CR, Grayson ML, Hansen P, Singh N, Theuretzbacher U, Magrini N, WHO Pathogens Priority List Working Group. 2018. Discovery, research, and development of new antibiotics: the WHO priority list of antibiotic-resistant bacteria and tuberculosis. Lancet Infect Dis 18:318–327. doi:10.1016/S1473-3099(17)30753-3 29276051

[3] He T, Wang R, Liu D, Walsh TR, Zhang R, Lv Y, Ke Y, Ji Q, Wei R, Liu Z, et al.. 2019. Emergence of plasmid-mediated high-level tigecycline resistance genes in animals and humans. Nat Microbiol 4:1450–1456. doi:10.1038/s41564-019-0445-2 31133751

[4] Barreto-Hernández E, Falquet L, Reguero MT, Mantilla JR, Valenzuela EM, González E, Cepeda A, Escalante A. 2013. Draft genome sequences of multidrug-resistant Acinetobacter sp. strains from Colombian hospital. Genome Announc 1:e00868–13. doi:10.1128/genomeA.00868-13 24285656 PMC3869318

[5] Chen C, Cui C-Y, Yu J-J, He Q, Wu X-T, He Y-Z, Cui Z-H, Li C, Jia Q-L, Shen X-G, Sun R-Y, Wang X-R, Wang M-G, Tang T, Zhang Y, Liao X-P, Kreiswirth BN, Zhou S-D, Huang B, Du H, Sun J, Chen L, Liu Y-H. 2020. Genetic diversity and characteristics of high-level tigecycline resistance tet(X) in Acinetobacter species. Genome Med 12:111. doi:10.1186/s13073-020-00807-5 33287863 PMC7722449

[6] D’Souza AW, Potter RF, Wallace M, Shupe A, Patel S, Sun X, Gul D, Kwon JH, Andleeb S, Burnham C-AD, Dantas G. 2019. Spatiotemporal dynamics of multidrug resistant bacteria on intensive care unit surfaces. Nat Commun 10:4569. doi:10.1038/s41467-019-12563-1 31594927 PMC6783542

[7] Chen S, Zhou Y, Chen Y, Gu J. 2018. fastp: an ultra-fast all-in-one FASTQ preprocessor. Bioinformatics 34:i884–i890. doi:10.1093/bioinformatics/bty560 30423086 PMC6129281

[8] Wick RR, Judd LM, Gorrie CL, Holt KE. 2017. Unicycler: resolving bacterial genome assemblies from short and long sequencing reads. PLoS Comput Biol 13:e1005595. doi:10.1371/journal.pcbi.1005595 28594827 PMC5481147

[9] Wood DE, Lu J, Langmead B. 2019. Improved metagenomic analysis with Kraken 2. Genome Biol 20:257. doi:10.1186/s13059-019-1891-0 31779668 PMC6883579

[10] Bortolaia V, Kaas RS, Ruppe E, Roberts MC, Schwarz S, Cattoir V, Philippon A, Allesoe RL, Rebelo AR, Florensa AF, et al.. 2020. ResFinder 4.0 for predictions of phenotypes from genotypes. J Antimicrob Chemother 75:3491–3500. doi:10.1093/jac/dkaa345 32780112 PMC7662176

[11] Naas T, Oueslati S, Bonnin RA, Dabos ML, Zavala A, Dortet L, Retailleau P, Iorga BI. 2017. Beta-lactamase database (BLDB) – structure and function. J Enzyme Inhib Med Chem 32:917–919. doi:10.1080/14756366.2017.1344235 28719998 PMC6445328

[12] Seemann T. 2014. Prokka: rapid prokaryotic genome annotation. Bioinformatics 30:2068–2069. doi:10.1093/bioinformatics/btu153 24642063

[13] Camacho C, Coulouris G, Avagyan V, Ma N, Papadopoulos J, Bealer K, Madden TL. 2009. BLAST+: architecture and applications. BMC Bioinformat 10:421. doi:10.1186/1471-2105-10-421 PMC2803857 20003500

[14] Carattoli A, Zankari E, García-Fernández A, Voldby Larsen M, Lund O, Villa L, Møller Aarestrup F, Hasman H. 2014. In silico detection and typing of plasmids using plasmidfinder and plasmid multilocus sequence typing. Antimicrob Agents Chemother 58:3895–3903. doi:10.1128/AAC.02412-14 24777092 PMC4068535

[15] Robertson J, Nash JHE. 2018. MOB-suite: software tools for clustering, reconstruction and typing of plasmids from draft assemblies. Microb Genom 4:e000206. doi:10.1099/mgen.0.000206 30052170 PMC6159552

[16] Ankenbrand MJ, Hohlfeld S, Hackl T, Förster F. 2017. AliTV—interactive visualization of whole genome comparisons. PeerJ Comput Sci 3:e116. doi:10.7717/peerj-cs.116

[17] Ma J, Wang J, Feng J, Liu Y, Yang B, Li R, Bai L, He T, Wang X, Yang Z. 2020. Characterization of three porcine Acinetobacter towneri strains co-harboring tet(X3) and bla_OXA-58_. Front Cell Infect Microbiol 10:586507. doi:10.3389/fcimb.2020.586507 33363052 PMC7758954

[18] Wang J, Wang Y, Wu H, Wang Z-Y, Shen P-C, Tian Y-Q, Sun F, Pan Z-M, Jiao X. 2020. Coexistence of bla_OXA-58_ and tet(X) on a novel plasmid in Acinetobacter sp. from pig in Shanghai China. Front Microbiol 11. doi:10.3389/fmicb.2020.578020 PMC7530245 33042094

[19] Zhang R, Dong N, Zeng Y, Shen Z, Lu J, Liu C, Huang Z, Sun Q, Cheng Q, Shu L, Cai J, Chan EW, Liu D, Chen G, Wang Y, Chen S. 2020. Chromosomal and plasmid-borne tigecycline resistance genes tet(X3) and tet(X4). Antimicrob Agents Chemother 64:e00674-20. 32816739 10.1128/AAC.00674-20 PMC7577139

[20] Cheng Y, Li Y, Yu R, Ma M, Yang M, Si H. 2022. Identification of novel tet(X3) variants resistant to tigecycline in Acinetobacter species. Microbiol Spectr 10:e0133322. doi:10.1128/spectrum.01333-22 36409072 PMC9784759

[21] Fang L-X, Chen C, Cui C-Y, Li X-P, Zhang Y, Liao X-P, Sun J, Liu Y-H. 2020. Emerging high‐level tigecycline resistance: novel tetracycline destructases spread via the mobile tet(X). Bioessays 42:e2000014. doi:10.1002/bies.202000014 32567703

[22] Chang D, Mao Y, Qiu W, Wu Y, Cai B. 2023. The source and distribution of tetracycline antibiotics in China: a review. Toxics 11:214. doi:10.3390/toxics11030214 36976979 PMC10052762

